# Profiling of MicroRNAs in Midguts of *Plutella xylostella* Provides Novel Insights Into the *Bacillus thuringiensis* Resistance

**DOI:** 10.3389/fgene.2021.739849

**Published:** 2021-09-08

**Authors:** Jie Yang, Xuejiao Xu, Sujie Lin, Shiyao Chen, Guifang Lin, Qisheng Song, Jianlin Bai, Minsheng You, Miao Xie

**Affiliations:** ^1^State Key Laboratory of Ecological Pest Control for Fujian and Taiwan Crops, Institute of Applied Ecology, Fujian Agriculture and Forestry University, Fuzhou, China; ^2^Joint International Research Laboratory of Ecological Pest Control, Ministry of Education, Fujian Agriculture and Forestry University, Fuzhou, China; ^3^Ministerial and Provincial Joint Innovation Centre for Safety Production of Cross-Strait Crops, Fujian Agriculture and Forestry University, Fuzhou, China; ^4^College of Life Sciences, Fujian Agriculture and Forestry University, Fuzhou, China; ^5^Division of Plant Sciences, University of Missouri, Columbia, MO, United States

**Keywords:** microRNAs, *Bacillus thuringiensis*, resistance, *Plutella xylostella*, differential expression analysis

## Abstract

The diamondback moth (DBM), *Plutella xylostella*, one of the most destructive lepidopteran pests worldwide, has developed field resistance to *Bacillus thuringiensis* (Bt) Cry toxins. Although miRNAs have been reported to be involved in insect resistance to multiple insecticides, our understanding of their roles in mediating Bt resistance is limited. In this study, we constructed small RNA libraries from midguts of the Cry1Ac-resistant (Cry1S1000) strain and the Cry1Ac-susceptible strain (G88) using a high-throughput sequencing analysis. A total of 437 (76 known and 361 novel miRNAs) were identified, among which 178 miRNAs were classified into 91 miRNA families. Transcripts per million analysis revealed 12 differentially expressed miRNAs between the Cry1S1000 and G88 strains. Specifically, nine miRNAs were down-regulated and three up-regulated in the Cry1S1000 strain compared to the G88 strain. Next, we predicted the potential target genes of these differentially expressed miRNAs and carried out GO and KEGG pathway analyses. We found that the cellular process, metabolism process, membrane and the catalytic activity were the most enriched GO terms and the Hippo, MAPK signaling pathway might be involved in Bt resistance of DBM. In addition, the expression patterns of these miRNAs and their target genes were determined by RT-qPCR, showing that partial miRNAs negatively while others positively correlate with their corresponding target genes. Subsequently, novel-miR-240, one of the differentially expressed miRNAs with inverse correlation with its target genes, was confirmed to interact with *Px017590* and *Px007885* using dual luciferase reporter assays. Our study highlights the characteristics of differentially expressed miRNAs in midguts of the Cry1S1000 and G88 strains, paving the way for further investigation of miRNA roles in mediating Bt resistance.

## Introduction

*Bacillus thuringiensis* (Bt), a class of spore-forming gram-positive bacterium that can produce different insecticidal crystal proteins (Cry and Cyt toxins), has been widely used as an entomopathogen for pest control (Wu et al., 2008; Raymond et al., 2010; Palma et al., 2014). The application of Bt toxins cannot only increase crop yields and bring substantial economic benefits, but also reduce environmental pollution caused by the abuse of chemical insecticides (Bravo et al., 2011). Bt Cry toxin has specific control efficiency on lepidopteran pests, and the mode of action involves toxin solubilization, proteolytic activation, interaction with midgut proteins of insects, formation of a pre-pore oligomeric structure, facilitation of the insertion into cell membrane, and creation of an ionic pore that kills midgut cells (Bravo et al., 2011). There are several resistance strategies taken by insects to counter Bt toxins, including mutations in receptor [e.g., cadherin, aminopeptidase N (APN) or ATP-binding cassette (ABC) transporters], alteration in Cry toxin activation and binding ability to gut membrane, sequestration of toxins by glycolipid moieties or esterases, and the elevation of insects immune responses (Pardo-Lopez et al., 2013). So far, more than 22 cases of field-evolved Bt resistance in several insect species have been documented, potentially reducing the control efficacy of Bt toxins toward agricultural pests (Janmaat and Myers, 2003; Tabashnik and Carrière, 2017, 2019; Calles-Torrez et al., 2019). Therefore, to delay the resistance evolution, it is urgent to understand the mechanisms causing Bt resistance and provide novel targets for pest control.

The diamondback moth (DBM), *Plutella xylostella*, a notorious pest of cruciferous crops, has caused severe economic losses globally. One of the main challenges of DBM control is its rapid evolution of resistance against a wide range of insecticides, including Bt-based products (Furlong et al., 2013). Considering its severe insecticide-resistance, short life cycle and host range expansion (Li et al., 2016), microRNA (miRNA)-mediated efficient and environmentally friendly approaches have been proposed to combat DBM (Vaschetto and Beccacece, 2019).

MiRNAs are a class of endogenous non-coding RNAs ranged from 19 to 24 nt in length and play crucial roles in the post-transcriptional regulation (Bartel, 2004; Cullen, 2004; Asgari, 2013). MiRNAs regulate gene expression by partially or completely binding their seed sequence region with the 3′ untranslated region (3′UTR) of corresponding target genes (Bartel, 2004). A large number of miRNAs have been identified from multiple insect species, participating in insect development (Liu et al., 2018, 2020b; Xu et al., 2019; Lim et al., 2020), reproduction (Lucas et al., 2015; He et al., 2016; Ling et al., 2017), behavior (Cristino et al., 2014; Chen and Rosbash, 2016; Niu et al., 2019), and host-pathogen interaction (Singh et al., 2014; Dubey et al., 2019; Wu et al., 2019). Recently, a growing body of evidence has illustrated the pivotal role of miRNAs in responses to environmental chemical and pathogen exposures, which attracts great interests in the field of insect toxicology. Insecticide resistance is predominantly caused by the rising metabolic detoxification of insecticides and declining in the target sites sensitivity of the insecticides (Liu, 2015). MiRNAs enhance insecticide resistance by negatively regulating the expression of detoxification-related genes or altering insect susceptibility to various kinds of insecticides. In *Culex pipiens pallens*, the pyrethroid-resistance is modulated by miR-92a via suppressing *CpCPR4* expression (Ma et al., 2017), miR-278-3p and miR-932 are also involved in the pyrethroid resistance by targeting *CYP6AG11* and *CpCPR5*, respectively (Lei et al., 2014; Liu et al., 2016). In addition, it has been reported that miR-13664 could interact with *CpCYP314A1* and subsequently regulate deltamethrin resistance (Sun et al., 2019). Another study confirms the significant role of miR-2∼13–71 cluster in deltamethrin resistance by regulating the expression of *CYP9J35* and *CYP325BG3* (Guo et al., 2017). In DBM, miR-7a and miR-8519 regulate chlorantraniliprole resistance via overexpressing *ryanodine receptor* (*RyR*) (Li X. et al., 2015). MiR-2b-3p and miR-14-5p are proposed to inhibit the detoxification pathways by suppressing the transcript levels of *CYP9F2* and *CYP307a1*, respectively (Etebari et al., 2018). MiR-189942 directly targets the *ecdysone receptor* (*EcR*) *isoform B* and increased the tolerance to fufenozide (Li X. et al., 2020). Besides, miR-998-3p is implicated in Bt resistance through targeting *ABCC2*, a putative receptor for Bt Cry1Ac toxin in three lepidopterans *P. xylostella, Helicoverpa armigera*, and *Spodoptera exigua* (Zhu et al., 2019). While these studies provide insight into the critical roles of miRNAs in regulating metabolic resistance to insecticides, most of them are focused on the chemical insecticide. The mechanism of miRNA-mediated Bt resistance still remains limited.

A previous study has profiled the dynamic expression patterns of miRNAs in response to Bt at different time courses using the whole body of DBM, indicating the potential role of miR-2b-3p in regulating insect immunity (Li S. et al., 2019). In the present study, we aimed to expand the numbers of identified miRNAs in specific tissues, i.e., midguts of the Bt resistant and susceptible DBM stains and explore the potential mechanisms of miRNA-mediated regulation in Bt resistance. The differentially expressed miRNAs in midguts of both strains were putatively linked to Bt resistance. One of the differentially expressed miRNAs, novel-miR-240, which showed an inverse correlation with its target genes, was then selected for dual luciferase assays to validate the interaction with its target genes. Our work is important for further understanding the potential roles of miRNAs in the evolution of Bt resistance in DBM field populations, providing novel insights for their putative applications in integrated pest management.

## Materials and Methods

### Insects Rearing

The insecticide-susceptible DBM strain (Geneva 88, G88) was originally collected from the New York State Agricultural Experiment Station in 1988 and maintained on an artificial diet without exposure to insecticide. A colony of DBM, which was established from a crucifer field in Florida in 1992, was used to investigate its resistance to *B. thuringiensis* subsp. *kurstaki* spray formula. Specifically, it was challenged with the transgenic broccoli expressing Cry1Ac toxin, and the survived moths were maintained as a Cry1Ac-R strain (Zhao et al., 2002). The Cry1Ac-R resistance strain was further selected with 1,000 μg/ml Cry1Ac protoxin in 2016 and then reared on an artificial diet without additional insecticide exposure for more than 50 generations. This strain was thereafter named as Cry1S1000 (Liu et al., 2020a). Both the G88 susceptible and Cry1S1000 resistance strains were reared with the same approach reported before (Xu et al., 2020). In brief, artificial diet and 10% honey solution were, respectively, used to feed larvae and adults at 25 ± 1°C, with a 16:8 h light: dark cycle and 65 ± 5% relative humidity.

### Sample Collection and Small RNA Library Construction

Fresh midguts dissected from 60 4th instar larvae of the Cry1S1000 and G88 strains were collected in separate tubes and the midgut contents were gently scraped off. The midgut samples were immediately immersed in liquid nitrogen and subsequently frozen in -80°C until further analysis. To generate a small RNA (sRNA) library, the miRNeasy Mini Kit (Qiagen, Germany) was used for total RNA extraction from the midgut samples. RNA concentration and purity were measured using the NanoDrop 2000 (Thermo Fisher Scientific, United States) and Agilent Bioanalyzer 2100 system (Agilent Technologies, United States), respectively. To generate sRNA libraries, 3 μg RNA per sample was used for adaptor ligation in both of 5′ and 3′ ends using the NEBNext Multiplex Small RNA Library Prep Kit for Illumina (NEB, United States). Then, the M-MuLV Reverse Transcriptase (RNase H^–^) was used for the first strand cDNA synthesis. The PCR amplification was performed and the fragments of 140∼160 bp were recovered and sequenced using the Illumina HiSeq 2500 sequencing platform (Illumina Inc., United States).

### Bioinformatic Analysis

To obtain the clean reads, low-quality reads, reads containing more than 10% unknown base N, without 3′ adaptor sequence, shorter than 18 nt, or longer than 30 nt and 3′ adapter reads were removed from the raw sequences, and the identical reads were collapsed in order to remove redundancy. After that, the trimmed sequences were aligned to Silva, GtRNAdb, Rfam, and Repbase databases to filter the ncRNAs including ribosomal RNA (rRNA), transfer RNA (tRNA), small nuclear RNA (snRNA), small nucleolar RNA (snoRNA), and repetitive RNAs. The remaining reads mapped to the reference genome^1^ with Bowie software were used for further miRNA identification. Known miRNAs were identified by aligning with mature sequences in miRBase (release22)^2^ with one mismatched allowed. For novel miRNAs identification, the miRDeep2 package was utilized to obtain potential precursor sequences. Prediction of novel miRNAs was completed based on the precusor stucture energy information and the distribution infromation of reads on precursor sequence (Friedlander et al., 2012).

### Expression Patterns of miRNAs Based on Transcripts per Million

To identify the miRNAs differentially expressed in the Cry1S1000 and G88 strains, transcripts per million (TPM) was used to estimate miRNA expression levels. The normalization formula was: normalized expression = (number of mapped reads for each miRNA/total number of mapped reads) × 10^6^. The DESeq2 algorithm (Love et al., 2014) was performed to estimate the differences in reads frequencies comparing the Cry1S1000 to G88 strains, and *p*-value was adjusted using the Benjamini–Hochberg false discovery rate (FDR) procedure (Puoliväli et al., 2020). Only those miRNAs with a log2 fold-change > 1.58 or < –1.58 in expression and a FDR ≤ 0.01 were determined to be significantly differentially expressed between the Cry1S1000 and G88 strains.

### Target Gene Prediction and Enrichment Analysis

To predict and analyze the potential target genes of differentially expressed miRNAs, miRanda (Rehmsmeier et al., 2004), and TargetScan (Lewis et al., 2005) algorithms with default parameters were employed. The DBM genome sequence (DBM-DB)^3^ was used as a reference and the coding sequence (CDS) was searched for predicting miRNA targets. To screen more reliable results, only those genes predicted by both algorithms were retained. The predicted miRNA targets were annotated based on NR, Swiss-Prot (Apweiler et al., 2004), Gene Ontology (GO) (Ashburner et al., 2000), COG (Tatusov et al., 2000), Kyoto Encyclopedia of Genes and Genomes (KEGG) (Kanehisa et al., 2004), KOG (Koonin et al., 2004) and Pfam databases using BLAST program with a cut-off *E*-value = 10^–5^. To further investigate the functions of the putative target genes, the software Database for Annotation Visualization and Integrated Discovery (DAVID)^4^ was used to conduct the GO enrichment consisted of biological processes, cellular components and molecular functions. Pathway analysis was based on the KEGG database that determine the pathways associated with the target genes of the differentially expressed miRNA. The DBM genome database was used as a background to determine the most enriched GO terms with a corrected *p*-value ≤ 0.05 as the threshold. The KEGG analysis was performed and a corrected *p*-value was set as 0.05 for identifying significantly enriched pathways within the predicted differentially expressed miRNA target dataset.

### Expression Patterns of miRNAs and Target Genes Based on RT-qPCR

Total RNA was extracted from midguts of 20 fourth instar larvae using a PF miRNA Isolation Kit (Omega, United States), and the concentration was measured by NanoDrop 2000 (Thermo Fisher Scientific, United States). The miRNA reverse transcription reactions were conducted using the miScript II RT kit (Qiagen, Germany), and the RevertAid First Strand cDNA Synthesis Kit (Thermo Fisher Scientific, United States) was used to synthesize the first-strand cDNA of target genes. Reverse transcriptase-quantitative PCR (RT-qPCR) reactions of miRNAs were performed using the miScript SYBR Green PCR kit (Qiagen, Germany) with miRNA-specific forward primers (Supplementary Table 1), and the RT-qPCR reactions of target genes were performed using the Eastep qPCR Master Mix (Promega, United States. All amplification reactions were performed on the Bio-Rad Realt-time PCR system (Bio-Rad, United States) with three technical repeats and three independent biological replicates. The normalized expression levels were calculated with 2^–ΔΔ*Ct*^ method using the internal references U6 snRNA for miRNAs normalization and *RPL32* for target genes normalization. The significance was analyzed using Student’s *t*-test with SPSS 25.0 program. All the reagents and kits used in the current study were applied according to the manufacturer’s instruction unless stated specifically.

### Dual Luciferase Reporter (DLR) Assay

Novel-miR-240 agomir (5′-UCCUCAAUAUCAUAUUCC UCGC-3′) and negative control (NC) agomir (5′-UUCUCCGAACGUGUCACGUTT-3′) were synthesized by Sangon Biotech (Shanghai, China). An agomir is a sequnce of double-strand RNA with special chemical modification, and it can mimic the function of miRNA and up-regulate the expression of endogenous miRNA. The luciferase reporter plasmids were constructed by cloning the wild type (WT) and the mutated (MUT) target sequences of *Px017590* and *Px007885* into the pmirGLO vector (Promega, United States). The fragments of *Px017590* and *Px007885* containing WT and MUT sequences were then confirmed by sequencing. To obtain high transfection effciency and low background expression, HEK293T cell line was used for the DLR assay. HEK293T cells were cultured in a 24-well plate and co-transfected with the reporter plasmid and the miRNA agomir or NC agomir using the Attractene Tranfection Reagent (Qiagen, Germany). Each well containing 1 μg reporter plasmid and 50 nM miRNA agomir or NC agomir. The activeties of the Firefly and Renilla luciferases were preformed by using a Dual-Glo Luciferase Assay System (Promega, United States) at 48 h post-transfection. Firefly luciferase activity was normalize to Renilla luciferase activity.

## Results

### Overview of the sRNA Libraries

To analyze the role of miRNAs in the Bt resistance of DBM, we performed the deep sequencing for identification and characterization of miRNAs in the larval midguts of the Cry1S1000 and G88 strains. Using the high-throughput sequencing, a total of 125,558,480 raw reads (≥17.6 million per library) were obtained. After removing low-quality reads and 3’ adaptor, only the reads ranging from 18 to 30 nt were kept. Clean reads from three Cry1S1000 libraries (15,964,505, 18,860,033, and 16,118,802) and three G88 libraries (12,107,465, 13,815,968, and 18,445,775) were obtained, respectively. The clean reads were aligned with Silva, GtRNAdb, Rfam, and Repbase databases and then mapped to the reference genome using the Bowtie package. A total of 9,200,300 mapped reads were retained and used to predict mature miRNAs in subsequent analyses (Table 1).

**TABLE 1:** sRNA libraries of the Cry1S1000 and G88 strains.

Group of reads*	**Number of reads**						**Total reads**
	**Cry1S1000-1**	**Cry1S1000-2**	**Cry1S1000-3**	**G88-1**	**G88-2**	**G88-3**	
Raw reads	21,847,848	22,213,713	20,886,851	17,688,151	19,614,812	23,307,105	125,558,480
Clean reads	15,964,505	18,860,033	16,118,802	12,107,465	13,815,968	18,445,775	95,312,548
Unannotated reads	7,404,038 (46.38%)	8,081,934 (42.86%)	6,681,832 (41.45%)	4,213,087 (34.8%)	6,724,759 (48.68%)	7,604,070 (41.22%)	40,709,720
rRNA	8,251,242 (51.68%)	10,487,087 (55.60%)	9,224,539 (57.23%)	76,575,610 (63.25%)	6,597,046 (47.75%)	10,448,645 (56.65%)	121,584,169
tRNA	307,827 (1.93%)	288,842 (1.53%)	210,446 (1.31%)	235,307 (1.94%)	492,090 (3.56%)	390,902 (2.12%)	1,925,414
snRNA	0 (0.00%)	0 (0.00%)	0 (0.00%)	0 (0.00%)	1 (0.00%)	1 (0.00%)	2
snoRNA	90 (0.00%)	183 (0.00%)	241 (0.00%)	140 (0.00%)	237 (0.00%)	211 (0.00%)	1,102
Repeat	1,308 (0.01%)	1,987 (0.01%)	1,744 (0.01%)	1,421 (0.01%)	1,835 (0.01%)	1,946 (0.01%)	10,241
Mapped reads	1,675,527 (22.63%)	1,796,235 (22.23%)	1,215,274 (18.19%)	1,058,410 (25.12%)	1,650,946 (24.55%)	1,803,908 (23.72%)	9,200,300
Known miRNA	57	59	57	53	67	60	76
Novel miRNA	289	288	264	249	327	321	361

*^∗^rRNA, ribosomal RNA; tRNA, transfer RNA; snRNA, small nuclear RNA; snoRNA, small nucleolar RNA.*

### Identification of Known and Novel miRNAs

**FIGURE 1:**
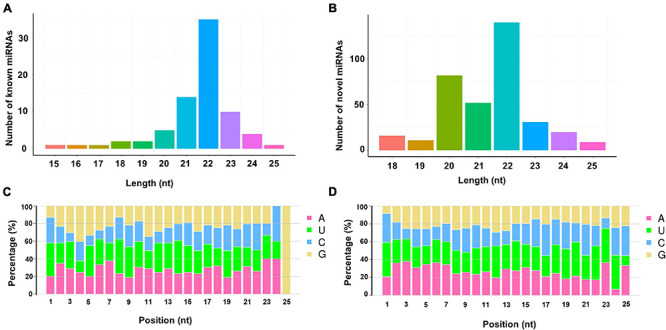
Lengths distribution and nucleotide bias of miRNAs identified in the sRNA libraries. **(A)** Lengths distribution of known miRNAs; **(B)** lengths distribution of novel miRNAs; **(C)** nucleotide bias of known miRNAs; **(D)** nucleotide bias of novel miRNAs.

Bioinformatic analysis was carried out to identify miRNAs expressed in the larval midguts of the Cry1S1000 and G88 strains. By searching against miRBase, 76 miRNAs in our libraries were identical to mature miRNA sequences and annotated as known miRNAs. Additionally, the miRDeep2 software package (Wen et al., 2012) was used to predict novel miRNAs by exploring the potential precursor sequences and estimating their randfold value. In total, 361 novel miRNAs were identified (Table 1). The length distribution of both known and novel miRNAs showed a peak at 22 nt, which is the standard size of animal miRNAs (Figures 1A,B). The first base preference of identified miRNAs was also investigated, and the result showed a dominant bias toward uracil (U). This bias was particularly evident in both known miRNAs (20 and 22 nt in length) and novel miRNAs (19 nt and 22 nt in length). Calculation of the miRNA nucleotide bias at each position demonstrated that U and A (adenine) were more common than C (cytosine) and G (guanine) in almost all positions, especially at base 14 (Figures 1C,D).

**FIGURE 2:**
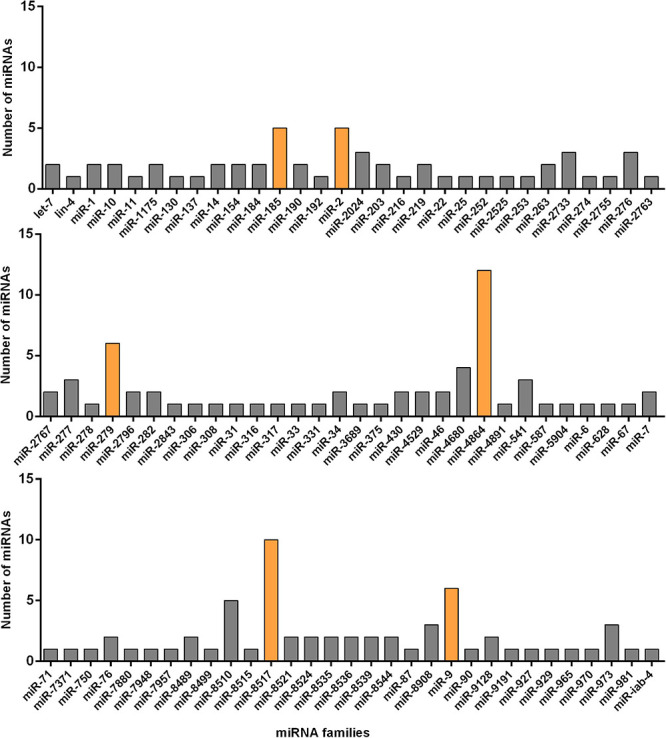
Number of known miRNAs expressed in the resistant (Cry1S1000) and susceptible (G88) strains of DBM in each of the miRNA families. The families with relatively more members are marked with orange color while others with gray.

Next, we classified 178 known and novel miRNAs into 91 families based on the sequence conservation across different species, while the remaining 259 miRNAs could not be classified into any families. Six main families, which hold relatively more members compared with other sets, were discovered. To be specific, 12 identified miRNAs belonged to the miR-4864 family and ten miRNAs belonged to the miRNA-8517 family. It was also estimated that the miR-279 and miR-9 families, respectively, contained six miRNAs, while the other two miRNA families (miR-2 and miR-185) were each represented by five miRNAs. In comparison, the remaining families were predicted to have less than five miRNA members each (Figure 2). All of the 178 identified miRNAs and their miRNA families were listed in Supplementary Table 2.

**FIGURE 3:**
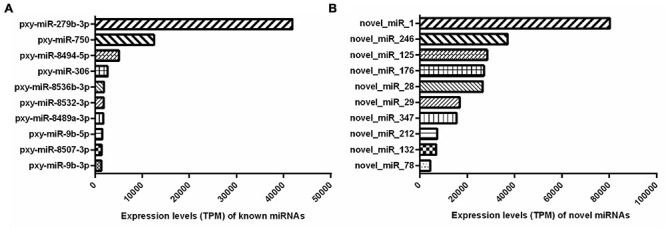
Top 10 abundantly expressed known **(A)** and novel **(B)** miRNAs in both the resistant (Cry1S1000) and susceptible (G88) strains. Average expression values of top 10 abundantly miRNAs are presented. TPM, transcripts per million.

In addition, the miRNA expression levels were assessed by TPM formula. The most abundant known miRNA was pxy-miR-279b-3p, followed by pxy-miR-750, pxy-miR-8494-5p, pxy-miR-306, and pxy-miR-8532-3p, while the five most highly expressed novel miRNAs were novel-miR-1, novel-miR-246, novel-miR-176, novel-miR-28, and novel-miR-132. Interestingly, the average expression value of the listed novel miRNAs was significantly higher than the known miRNAs. The most abundant known and novel miRNAs in the libraries were represented in Figure 3 and the details of all the identified miRNAs were listed in Supplementary Table 3.

### Differentially Expressed miRNAs in the Cry1S1000 and G88 Strains

**FIGURE 4:**
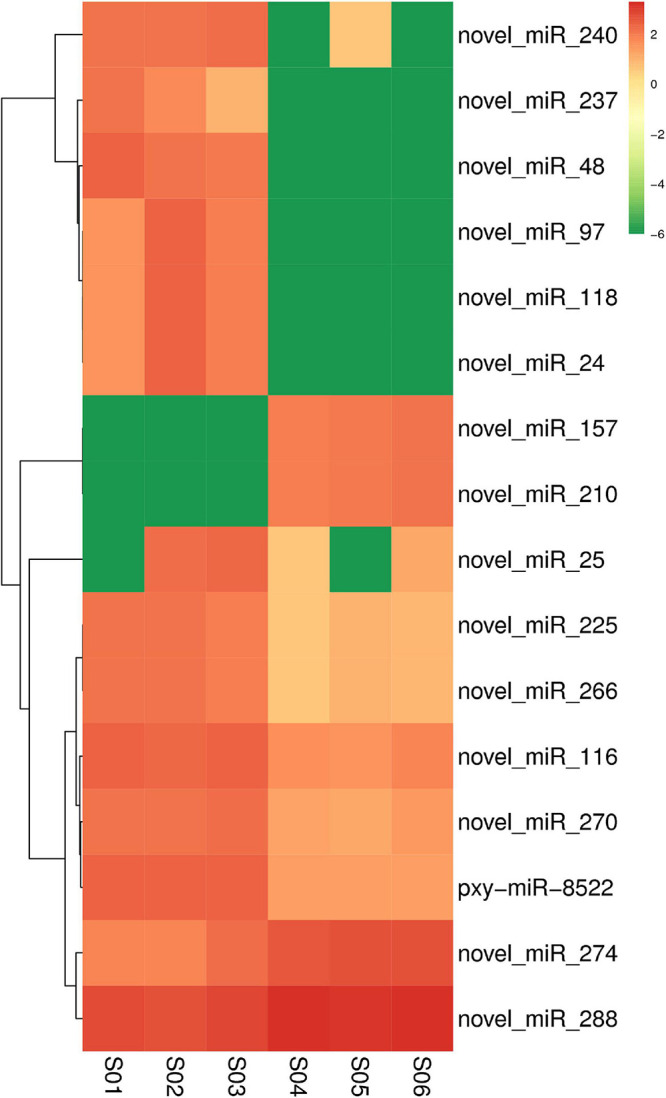
Cluster analysis diagram showing the differentially expressed miRNAs in the resistant (Cry1S1000) and susceptible (G88) strains. Clustering was performed with log_10_ (TPM + 1E-6) values. Samples of the G88 strain, S01–S03; samples of the Cry1S1000 strain, S04–S06. Columns represent different samples, while rows indicate different miRNAs. Red blocks represent the higher expressed miRNAs, green blocks represent the lower expressed miRNAs.

The expression levels of known and novel miRNAs were compared after normalization by TPM formula described above. Twelve differentially expressed miRNAs including nine down-regulated and three up-regulated miRNAs were found in the Cry1S1000 strain, compared to the G88 strain. In these miRNAs, novel-miR-210 and novel-miR-48 were predicted to show the strongest up-regulation and down-regulation trends, respectively (with the highest Log2-fold change value among listed miRNAs) (Table 2). Additionally, novel-miR-97 and novel-miR-237 were classified into the miR-973 and the miR-587 families, respectively, while others did not belong to any known miRNA family. Furthermore, the cluster analysis was performed to investigate the expression patterns of 12 differentially expressed miRNAs. Among all tested miRNAs, novel-miR-240, novel-miR-237, novel-miR-48, and novel-miR-97 showed significantly lower expression levels in the Cry1S1000 strain than in the G88 strain, while novel-miR-210 displayed a relatively higher expression level in the Cry1S1000 strain (Figure 4). It was noted that novel-miR-118 and novel-miR-24 shared the same sequence with novel-miR-97, novel-miR-157 shared the same sequence with novel-miR-210, and the sequence of novel-miR-266 was the same with novel-miR-225. Thus, these miRNAs sharing the same sequences were combined and, respectively, named as novel-miR-97, novel-miR-210, and novel-miR-225 hereafter.

**TABLE 2:** Differentially expressed miRNAs in the Cry1S000 and G88 strains.

**sRNA**	**Sequence (5′–3′)**	**Potential miRNA**	***E*-value**	**Length**	**miRNA family**	**Log2-fold change**	**Regulatory trend**
Novel_miR_210	uugugacguaggauugucaaua	Pma-miR-212-5p		22	–	3.970	Up
Novel-miR-274	ucagucucuguauucucccuuca	Pxy-miR-8529		23	–	2.030	Up
Novel-miR-288	cuaauccuccauagacucuagauu	Pxy-miR-8505 Gga-miR-7448-5p		24	–	1.624	Up
Novel-miR-48	uagcaccguagcauugaagu	*		20	–	–4.133	Down
Novel-miR-240	uccucaauaucauauuccucgc	*		22	–	–3.855	Down
Novel-miR-97	ccuagcuaguaauuaaucaucga	Prd-miR-60-3p		23	miR-973	–3.832	Down
Novel-miR-25	cagagaaaccugaccccccucc	*		22	–	–2.800	Down
Pxy-miR-8522	auuugccgaagguucugauacc			22	–	–2.795	Down
Novel-miR-237	uuuccaccagagaugugcuaug	*		22	miR-587	–2.787	Down
Novel-miR-225	uuaaaucgagauuugacacu	*		20	–	–2.750	Down
Novel-miR-270	aaacauccauguaaacauccuga	Ame-miR-9864-5p	3.0	23	–	–2.301	Down
Novel-miR-116	uaccuaagcaggaacuguuc	*		20	–	–1.957	Down

*Potential miRNA, sequences of differentially expressed miRNAs were similar to those in other species but differ in some nucleotide positions. ^∗^Means miRNA sequences were not found in other species in miRbase (V22) with E-value cutoff < 3.*

### Target Gene Prediction of the Differentially Expressed miRNAs

A total of 7,647 target genes of the miRNAs identified in our study were predicted by miRanda and TargetScan, of which 7,485 target genes were annotated with NR, Swiss-Prot, GO, COG, KEGG, KOG, and Pfam databases (Finn et al., 2014). To further explore the roles of the differentially expressed miRNAs, 44 genes potentially targeted by these 12 differentially expressed miRNAs were selected and represented in Table 3. Among them, the number of predicted target genes for each miRNA ranged from zero to 27, which implying a complex regulatory network between miRNAs and their target genes. Most miRNAs had multiple target genes. Conversely, some of the genes were also modulated by multiple miRNAs. Additionally, the annotation using the DBM-DB database showed that these putative transcripts were likely involved in multiple biological processes. Some of the differentially expressed miRNAs were predicted to regulate genes encoding chitinase A1, cuticle protein 6, transporting P-type ATPase, alyl-tRNA synthetase and cell wall protein, which might be closely related to the defense of external toxicant, energy transportation and metabolism and likely be involved in the insect immunity and insecticide resistance.

**TABLE 3:** Putative target genes of differentially expressed novel miRNAs.

**miRNA**	**Target gene**	**Annotation of target genes with DBM-DB**
Novel-miR-210	*Px006169*	–
	*Px000808*	Nesprin-1
Novel-miR-274	*Px013363*	Exostosin-2
	*Px012927*	Exocyst complex component 1
	*Px013839*	Carnitine O-palmitoyltransferase 1, liver isoform
	*Px007475*	Valyl-tRNA synthetase
	*Px014239*	Mannosyl-oligosaccharide glucosidase
	*Px009012*	Midasin
	*Px008940*	Moesin/ezrin/radixin homolog 2
	*Px008286*	Valyl-tRNA synthetase
	*Px011169*	Nephrin
	*Px002074*	Mycosubtilin synthase subunit C
	*Px002344*	S phase cyclin A-associated protein in the endoplasmic reticulum
	*Px001355*	Putative cation-transporting P-type ATPase
	*Px016226*	Moesin/ezrin/radixin homolog 2
	*Px006958*	Importin-7
	*Px002066*	Protogenin
	*Px008303*	Spondin, N-terminal
	*Px002006*	Polypeptide N-acetylgalactosaminyltransferase 5
	*Px001769*	Ubiquitin carboxyl-terminal hydrolase calypso
	*Px006107*	Interleukin-1 receptor accessory protein-like 1-B
	*Px016576*	E3 ubiquitin-protein ligase HERC2
	*Px010440*	GAS2-like protein 1
	*Px005898*	Putative uncharacterized protein
	*Px004830*	GG20930
	*Px002366*	MAGUK p55 subfamily member 6
	*Px009200*	Dynein heavy chain 3, axonemal
	*Px006757*	Cell wall protein, putative
	*Px008634*	Putative uncharacterized protein
Novel-miR-288	*Px016171*	Prolyl endopeptidase
Novel-miR-48	*Px011234*	Nose resistant to fluoxetine protein 6
	*Px006256*	Lachesin
	*Px004597*	Thyrotropin-releasing hormone receptor
Novel-miR-240	*Px003379*	Dynein heavy chain, cytoplasmic
	*Px017590*	Alsin
	*Px007885*	Discoidin domain-containing receptor 2
Novel-miR-25	*Px014091*	Leucine-rich PPR motif-containing protein, mitochondrial
	*Px015953*	Chitinase A1
	*Px002493*	Carbonic anhydrase 2
	*Px000596*	Cuticle protein 6
	*Px005729*	Monocarboxylate transporter 12
Novel-miR-237	*Px010315*	Bromodomain adjacent to zinc finger domain protein 2B
	*Px000280*	Elongation of very long chain fatty acids protein 7
	*Px016697*	Putative L-ribulose-5-phosphate 3-epimerase sgbU

### GO Enrichment and KEGG Pathway Analysis of Target Genes of Differentially Expressed miRNAs

**FIGURE 5:**
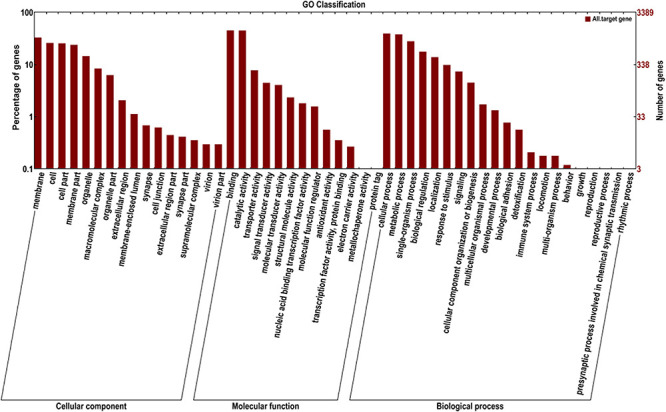
Gene ontology (GO) annotation of the genes targeted by identified miRNAs. The X-axis shows the GO annotation, and the Y-axis shows the ordinate left in the percentage of genes.

**FIGURE 6:**
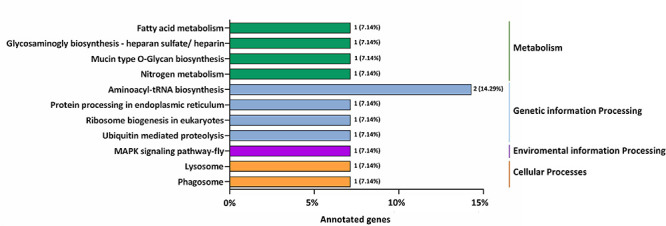
The most enriched KEGG pathways based on the genes targeted by differentially expressed miRNAs in the resistant (Cry1S1000) and susceptible (G88) strains. The X-axis shows the number of genes annotated to the pathway and the ratio of the number of annotated genes to the total genes annotated, and the Y-axis shows the pathway names. The different color of column indicates different type of KEGG pathway.

GO annotation enrichment was performed to evaluate the presumptive functions of target genes of differentially expressed miRNAs (Figure 5). GO term analysis for these target genes revealed seven cellular components, five molecular processes, and seven different biological processes, among which cellular process (GO:0009987), metabolic process (GO:0008152), single-organism process (GO:0044699), and biological regulation (GO:0065007) were dominant. Additionally, most of the target genes were classified in membrane (GO:0016020), binding (GO:0005488), and catalytic activity (GO:0003824), implying the putative roles of these differentially expressed miRNAs in transmembrane transport and translation. A KEGG pathway analysis was also conducted to elucidate the biological interpretation of genes targeted by differentially expressed miRNAs (Figure 6). The result exhibited that *Px008940* and *Px016226* were enriched in the Hippo signaling pathway, *Px007475* and *Px008286* were enriched in aminoacyl-tRNA biosynthesis and *Px006958* was enriched in MAPK signaling pathway, which were associated with energy metabolism in insects. In addition, the remaining target genes were enriched in the metabolic pathways such as ubiquitin mediated proteolysis, ribosome biogenesis in eukaryotes, protein processing in endoplasmic reticulum and fatty acid metabolism (Supplementary Table 4).

### RT-qPCR Validation of Differentially Expressed miRNAs and Their Putative Target Genes

**FIGURE 7:**
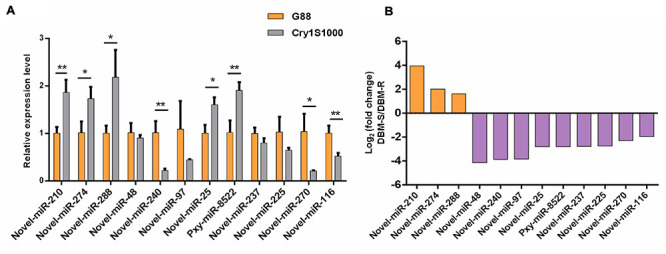
Relative expression levels of differentially expressed miRNAs in midguts of the resistant (Cry1S1000) and susceptible (G88) strains by RT-qPCR **(A)**, and up-/down-regulated expression of the miRNAs based on Illumina sequencing **(B).** The expression levels of miRNAs were normalized by U6. Statistical significance was analyzed using one-way ANOVA. The asterisks represent significance, where two asterisks indicate *p* < 0.01, and one asterisk indicates *p* < 0.05. **(B)** The orange column above the X-axis shows up-regulated miRNAs, while the purple column below the X-axis shows down-regulated miRNAs based on Illumina sequencing.

To further analyze the actual expression patterns of miRNAs identified above, RT-qPCR was perfomed to estimated the relative expression patterns of the 12 differentially expressed miRNAs (one known and 11 novel) in midguts of the Cry1S1000 and G88 strains, respectively (Figure 7A). Five miRNAs (novel-miR-210, novel-miR-274, novel-miR-288, novel-miR-25, and pxy-miR-8522) represented significantly higher expression levels in the Cry1S1000 strain than in the G88 strain, while the expression levels of other seven miRNAs (novel-miR-48, novel-miR-240, novel-miR-97, novel-miR-237, novel-miR-225, novel-miR-270, and novel-miR-116) were relatively lower in the Cry1S1000 strain, whereas only novel-miR-240, novel-miR-270, and novel-miR-116 exhibited a siginificant difference. Additionally, novel-miR-48, novel-miR-240, novel-miR-97, and novel-miR-237 were consistent with those predicted with TPM, whereas others presented opposite trends (Figure 7B).

**FIGURE 8:**
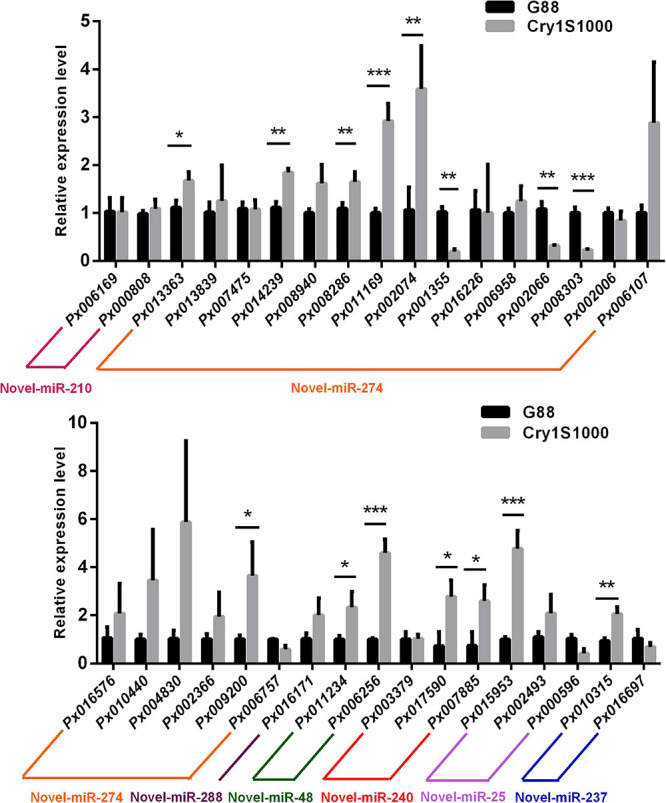
Relative expression levels of target genes in midguts of the resistant (Cry1S1000) and susceptible (G88) strains based on RT-qPCR. The expression levels of target genes were normalized by *RPL32*. Statistical significance was analyzed using one-way ANOVA. The asterisks represent significance, where three asterisks indicate *p* < 0.001, two asterisks indicate *p* < 0.01, and one asterisk indicates *p* < 0.05.

The potential target genes of differentially expressed miRNAs were investigated with two miRNA targets prediction algorithms, miRanda and TargetScan. A total of 34 target genes were profiled using RT-qPCR. Twelve target genes (*Px013363*, *Px014239*, *Px008286*, *Px011169*, *Px002074*, *Px009200*, *Px011234*, *Px006256*, *Px017590*, *Px007885*, *Px015953*, and *Px010315*) were significantly induced in the Cry1S1000 strain, among which *Px006256* and *Px015953* were siginificantly up-regulated for more than 4 times, while others were increased for 1∼3 times. Moreover, only four target genes (*Px001355*, *Px002066*, *Px008303*, and *Px000596*) showed the significantly lower expression levels in the Cry1S1000 strain compared to those in the G88 strain. The expression levels of *Px017590* and *Px007885* exhibited opposite trends with their corresponding novel-miR-240. But there was no predicted target genes for the differentially expressed novel-miR-270 and novel-miR-116 (Figure 8).

### DLR Validation of the Interaction Between Novel-miR-240 and Its Target Genes

**FIGURE 9:**
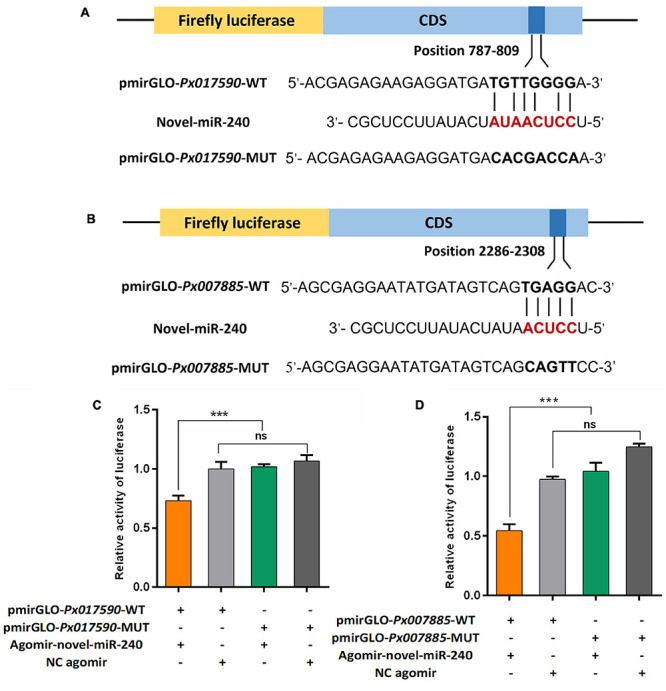
Dual luciferase reporter assay confirmed the interaction between novel-miR-240 and its target genes in vitro. **(A)** Predicted target sites of novel-miR-240 in the CDS of *Px017590*. **(B)** Predicted target sites of novel-miR-240 in the CDS of *Px007885*. WT, wild-type sequence; MUT, mutant sequence. **(C)** The interaction between novel-miR-240 and *Px017590*. **(D)** The interaction between novel-miR-240 and *Px007885*. Statistical significance was analyzed using one-way ANOVA followed by Tukey’s multiple comparisons. The three asterisks indicate *p* < 0.001 and “ns” represents no significance.

DLR assay was used to assessed whether the novel-miR-240 could regulated the expression of *Px017590* and *Px007885* by acting on their predicted binding sites. The target sequences (∼40 bp DNA fragments) containing the binding sites in the CDS of *Px017590* and *Px007885* were sythesized and cloned into the pmirGLO vector (pmirGLO-*Px017590*-WT and pmirGLO-*Px007885*-WT), respectively (Figures 9A,B). When novel-miR-240 agomir were co-transfected with pmirGLO-*Px017590*-WT or pmirGLO-*Px007885*-WT in HEK293T cells, the normalized firefly luciferase activity were siginificantly declined by 30 or 50% compared to the NC agomir control. Furthermore, we also designed the mutant fragments by altering the bases in the seed binding regions of *Px017590* and *Px007885* (pmirGLO-*Px017590*-MUT and pmirGLO-*Px007885*-MUT). As expected, the luciferase reporter activity was not affected by novel-miR-240 agomir, when it was co-transfected with pmirGLO-*Px017590*-MUT or pmirGLO-*Px007885*-MUT in HEK293T cells (Figures 9C,D). These results revealed that novel-miR-240 could regulate the expression of *Px017590* and *Px007885* by binding to their seed regions and therfore inhibiting mRNA translation.

## Discussion

MiRNAs, a class of endogenous non-coding RNAs, are considered to be modulators of gene expression at the post-transcriptional level (Lee et al., 2004). In recent years, with the development of the *in silico* miRNA identification platform such as mirMachine (Cagirici et al., 2021), miRanda (Rehmsmeier et al., 2004) and RNAhybrid (Mohebbi et al., 2021), many miRNAs have been characterized from multiple organisms including plants, vertebrates and invertebrates following the integration of various tools in bioinformatics, genetics, molecular biology and biochemistry. In insects, miRNAs played indispensable roles in development (Shen et al., 2020), behavior (Niu et al., 2019), immunity (Li R. et al., 2019), host-virus interaction (Singh, 2020), and resistance to multiple insecticides (Zhu et al., 2019; Li X. et al., 2020). In addition, the high-quality genome offers the opportunity to explore the putative biological function of identified miRNAs (Robertson et al., 2018). DBM is a notorious agriculture pest, understanding the roles played by miRNAs in insecticides resistance can provide a significant theoretical basis for finding novel targets for pest control.

Here, we successfully constructed sRNA libraries from Bt resistant and susceptible strains, and identified a total of 437 miRNAs including 76 known and 361 novel miRNAs belonging to 91 miRNA families. Importantly, 12 differentially expressed miRNAs (three up and nine down) were revealed in the Cry1S1000 strain compared to the G88 strain. We also predicted the putative target genes for each of these 12 differentially expressed miRNAs, and the target genes were enriched in 43 GO terms based on GO and KEGG pathway analyses. Furthermore, the expression patterns of these differentially expressed miRNAs and their potential target genes were investigated with RT-qPCR. Finally, the dual luciferase assay was carried out to confirm the interaction between novel-miR-240 and its target genes *Px017590* and *Px007885*.

From the identified miRNAs, a member of the miR-279 family, miR-279b-3p, was listed as the most abundant miRNA in our sRNA libraries. Previously, it has been found that miR-279, another member of the miR-279 family, was also listed as an abundantly expressed miRNA in DBM larvae after destruxin A injection and predicted to regulate the immunity-related genes (Shakeel et al., 2018). MiR-306, a common abundantly expressed miRNA identified in our study, has been shown to associated with Bt Cry1Ab resistance in *Ostrinia furnacallis* (Xu et al., 2015).

Additionally, a total of 78 known and novel miRNAs were classified into 91 miRNA families. MiRNAs sharing the same seed region were classified as a miRNA family, which likely indicated that they targeted the same genes and performed similar biological functions. In the current study, novel-miR-133 and novel-miR-250 were classified into the *let-7* family, which is functionally conserved from insects to humans and participated in multiple biological processes. In insects, modulating the abundance of *let-7* altered the tolerance of *Myzus persicae nicotianae* to nicotine (Peng et al., 2016). *Let-7* was also required in many developmental processes, such as sleep homeostasis (Goodwin et al., 2018), age-dependent behavioral changes (Behura and Whitfield, 2010), developmental transitions of egg hatching, molting, pupation, adult eclosion (Zhang et al., 2020) and the regulation of ecdysone levels (Liu et al., 2007).

Novel-miR-93 and novel-miR-125 were classified into the miR-14 family, which is conserved in insects and is closely related to olfactory and chemoreception (Shan et al., 2020). Another study showed that miR-14-3p, another member of the miR-14 family, could tightly control the ecdysone signaling pathway by regulating *EcR* and *E75* (Luo et al., 2020). A total of five miRNAs (novel-miR-142, novel-miR-214, novel-miR-219, novel-miR-222, and novel-miR-253) identified in our study belong to the miR-2 family. The conserved miR-2 family was an invertebrate-specific family of miRNAs and involved in insect metamorphosis (Lozano et al., 2015), oogenesis (Song et al., 2019) neural development (Marco et al., 2012), as well as deltamethrin resistance in *Cx. pipiens pallens* (Guo et al., 2017).

Pxy-miR-252 belonged to the miR-252 family. It has been found that miR-252 could directly repress the *mbt* expression to control the developmental growth of *Drosophila* (Lim et al., 2019) and was also involved in dengue virus replication in *Aedes albopictus* (Yan et al., 2014). Pxy-miR-274 was classified into the miR-274 family. Inhibition of miR-274-3p could facilitate *B. mori* cytoplasmic polyhedrosis virus (BmCPV) replication (Wu et al., 2017). Novel-miR-78 was classified into the miR-276 family. The miR-276 family might be involved in the spirotetramat resistance of *Aphis gossypii* Glover (Wei et al., 2016) and the dengue virus (DENV) replication in C6/36 cells (Su et al., 2019). Pxy-miR-277 belonged to the miR-277 family, which was conserved in insects and participated in multiple physiological processes such as restoring immune homeostasis of the IMD pathway (Li R. et al., 2020), controlling metamorphosis (Shen et al., 2020), lipid metabolism and reproduction (Ling et al., 2017). MiR-277-3p was also a part of the molecular toolkit regulating reproductive diapause in *Culex pipiens* (Meuti et al., 2018) and *Helicoverpa zea* (Reynolds et al., 2019). Hence, we speculate that the pxy-miR-277 might be invloved in diverse biological processes in DBM and indirectly affect detoxification of Bt Cry toxin. Pxy-miR-279a, pxy-279b-5p, and pxy-279-3p were classified to the miR-279 family. It has been reported that miR-279 was essential to maintain circadian rhythm (Sun et al., 2015) and reproductive responses by females to male sex peptide in *D. melanogaster* (Fricke et al., 2014). So, pxy-miR-279a, pxy-279b-5p, and pxy-279-3p might play the similar roles with miR-279 in insects. Pxy-miR-306 and pxy-miR-308 belonged to miR-306 and miR-308 families, respectively. MiR-279, miR-306, and miR-308 were also predicted to be involved in the Destruxin A-responsive immunity process in DBM (Shakeel et al., 2018), indicating that these miRNA families might be closely associated with Bt resistance. Novel-miR-21 was classified into the miR-317 family, and miR-317 was found to be involved in the process of pupation in *B. dorsalis* (Zhang et al., 2020). In *Drosophila*, miR-87 was an important regulator of dendrite regeneration (Kitatani et al., 2020), so we speculated that novel-miR-199 as a member of the miR-87 family, might paly the similar roles in DBM. In summary, these miRNA families identified in the current study were widely involved in diverse biological processes in insects, some of the miRNA families also took part in insect immunity. Investigating the functions of these conserved miRNA families and their representative members will provide a better understanding of miRNA-mediate post-transcriptional regulation in insects.

It has been previously shown that the differentially expressed miRNAs among the susceptible and resistant strains might be related to insecticides resistance in DBM. For instance, the expression of miR-276-5p and miR-8530-5p were 2–3 times higher in the chlorantraniliprole resistant DBM strain compared to the susceptible strain, indicating that these two miRNAs likely participated in the response of DBM to chlorantraniliprole (Zhu et al., 2017). The expression level of miR-278-3p was 4.2 fold lower in the deltamethrin-resistant strain (DR-strain), which induced the pyrethroid resistance in *Cx. pipiens pallens* (Lei et al., 2014). The other two conserved miRNAs, miR-932 and miR-285 were estimated to be 1.8- and 2.8-fold higher, respectively, in the DR-strain and were involved in pyrethroid resistance (Liu et al., 2016; Tian et al., 2016). In this study, the expression levels of novel-miR-240, novel-miR-270 and novel-miR-116 in the resistant Cry1S1000 strain were significantly lower than that in the susceptible G88 strain, other miRNAs such as novel-miR-210, novel-miR-274, novel-miR-288, novel-miR-25, and pxy-miR-8522 represented significantly higher expression levels in the resistant Cry1S1000 strain, and novel-miR-48, novel-miR-97, novel-miR-237, and novel-miR-225 exhibited no significant difference between these two strains. Moreover, the differentially expressed novel-miR-240, novel-miR-270, and novel-miR-116 did not belong to any known miRNA family, indicating that there was no miRNA shared the same seed regions or derived from the same arm of the stem-loop with these two miRNAs (Meyers et al., 2008). While novel-miR-270 had a similar sequence with ame-miR-9864-5p, suggesting that they may be the same miRNA in different species and play similar roles in insects. Our results suggested that the expression of miRNAs varies among different strains, and these differentially expressed miRNAs may regulate detoxification to Bt Cry1Ac toxin in a complicated manner by targeting detoxification-related genes.

It should be noted that novel-miR-240 represents down-regulated expression in the Cry1S1000 strain relative to the G88 strain, whereas its target genes *Px017590* and *Px007885* were up-regulated in the Cry1S1000 strain. The DLR assay results implied the involvement of novel-miR-240 in the regulation of *Px017590* and *Px007885*, indicating that this miRNA might modulate Bt resistance of DBM via negatively regulating its target genes.

Furthermore, GO annotation exhibited that most of the predicted target genes of differentially expressed miRNAs were enriched in biological process (cellular process, metabolism process, single-organism process, and biological regulation), cellular component (membrane, cell, cell part, and membrane part) and molecular function (binding and catalytic activity), which might be related to the insect immunity and the metabolism to the Bt Cry1Ac toxin. The target genes of differentially expressed miRNAs were mostly enriched in metabolic-related pathways such as Hippo signaling pathway, minoacyl-tRNA biosynthesis and MAPK signaling pathway. And in a previous study, MAPK signaling pathway was found to lead to Bt Cry1Ac resistance by mediating differential expression of APN and other midgut genes in DBM (Guo et al., 2020). The DBM-DB database was used for the annotation of target genes. *Px015953* and *Px000596*, the target genes of novel-miR-25, were predicted to be associated with chitinase and cuticle protein, which may be involved in the process of DBM combating the exogenous Bt Cry1Ac toxin.

Chitinase, an insect molting enzyme, is a potential biopesticide that degrades chitin to low molecular weight, soluble and insoluble oligosaccharides. Previous research has shown that chitinases could degrade the vital structures of insects such as their peritrophic membranes and cuticles, thus facilitating the entry of the pathogens into the tissues of susceptible insects (Brandt et al., 1978). Chitinases also facilitated the penetration of host cuticle by entomopathogenic fungi. Analysis of the top 20 differentially expressed genes showed that the chitinase ChiA2 was up-regulated after *Beauveria bassiana* infected non-natural hosts such as *H. armigera* and *Clanis bilineata* (Liu et al., 2021). These chitinase-related genes indicated that when entomopathogenic fungi transfer to non-original hosts, the fungi changed the metabolic response of hosts and used the novel infection strategies to break the barrier of different cuticle chitin components to better adaption to new hosts. Therefore, the chitin based bioformulation may act as an effective pesticide against Lepidopterous pests. A prior work revealed that the expression of cuticle proteins were siginificantly induced at 6 h post-treatment of Cry1Ac toxin (Qin et al., 2021). Genes encoding cuticle proteins were induced during *B. mori* bidensovirus (BmBDV) infection (Sun et al., 2020). The down-regulation of some *Cardinium*-responsive miRNAs enchanced the cuticle proteins in whitefly *Bemisia tabaci* (Gennadius) biotype Q and modulated insect reproduction and growth (Li et al., 2018). In addition, RNA-seq of *Cryptolestes ferrugineus* individuals from phosphine susceptible and resistant populations revealed the significantly increased expression levels of nine cuticular protein genes in the resistance population, demonstrating that the cuticular protein genes might be related to phosphine resistance (Chen et al., 2021). In the current study, the target genes of identified differentially expressed miRNAs, especially the chitinase and cuticle protein, might involved in the insects immune response and the detoxification of xenobiotics including the Bt Cry1Ac toxin. Although the RT-qPCR results showed that some of the identified differentially expressed miRNAs exhibited positively correlate with their potential targets, the target genes of these differentially expressed miRNAs were still worthy for further investigation, where functional characterizations of the target genes will provide novel insights of insects immunity and detoxification mechanisms.

## Conclusion

In the present study, we identified 76 known and 361 novel miRNAs from midguts of the Bt resistant and susceptible DBM strains. Among the identified miRNAs, 178 were classified into 91 miRNA families, and the potential functions of these miRNA families have also been illustrated. Expression of 12 differentially expressed miRNAs and their potetial target genes were profiled, revealing that these miRNAs may play vital roles in detoxicating Bt Cry1Ac toxins by regulating their corresponding target genes. Moreover, the interaction between novel-miR-240 and its target genes *Px017590* and *Px007885* have been confirmed by the DLR assay. However, further experiments are still required to demonstrate the functional pathways which the differentially expressed miRNAs and their target genes are involved in. Our study contributes to the field for further investigation of the miRNA-mediated gene regulation at the post-transcriptional level in insect resistance and the RNAi-based pest management.

## Data Availability Statement

The datasets presented in this study can be found in online repositories. The names of the repository/repositories and accession number(s) can be found below: NCBI SRA; PRJNA754494.

## Author Contributions

MX and MY conceived and designed the study. JY performed the experiments and analyzed the data with the help of GL, SC, and JB. JY wrote the first draft of the manuscript. XX, SL, MX, MY, and QS participated in manuscript drafted and modification. All authors reviewed and approved the final manuscript.

## Conflict of Interest

The authors declare that the research was conducted in the absence of any commercial or financial relationships that could be construed as a potential conflict of interest.

## Publisher’s Note

All claims expressed in this article are solely those of the authors and do not necessarily represent those of their affiliated organizations, or those of the publisher, the editors and the reviewers. Any product that may be evaluated in this article, or claim that may be made by its manufacturer, is not guaranteed or endorsed by the publisher.

## Footnotes

1
https://www.ncbi.nlm.nih.gov/genome/?term=Plutella+xylostella

2
http://www.mirbase.org/

3
http://59.79.254.1/dbm/

4
https://david.ncifcrf.gov/

## References

[B1] Apweiler R. Bairoch A. Wu C. H. Barker W. C. Boeckmann B. Ferro S. (2004). UniProt: the Universal Protein knowledgebase. *Nucleic Acids Res.* 32 D115–D119. 10.1093/nar/gkh131 14681372 PMC308865

[B2] Asgari S. (2013). MicroRNA functions in insects. *Insect Biochem. Mol. Biol.* 43 388–397. 10.1016/j.ibmb.2012.10.005 23103375

[B3] Ashburner M. Ball C. A. Blake J. A. Botstein D. Butler H. Cherry J. M. (2000). Gene ontology: tool for the unification of biology. The Gene Ontology Consortium. *Nat. Genet.* 25 25–29. 10.1038/75556 10802651 PMC3037419

[B4] Bartel D. P. (2004). MicroRNAs: genomics, biogenesis, mechanism, and function. *Cell* 116 281–297. 14744438 10.1016/s0092-8674(04)00045-5

[B5] Behura S. K. Whitfield C. W. (2010). Correlated expression patterns of microRNA genes with age-dependent behavioural changes in honeybee. *Insect Mol. Biol.* 19 431–439. 10.1111/j.1365-2583.2010.01010.x 20491979

[B6] Brandt C. R. Adang M. J. Spence K. D. (1978). The peritrophic membrane: ultrastructural analysis and function as a mechanical barrier to microbial infection in *Orgyia pseudotsugata*. *J. Invertebr. Pathol.* 32 12–24. 10.1016/0022-2011(78)90169-6

[B7] Bravo A. Likitvivatanavong S. Gill S. S. Soberón M. (2011). *Bacillus thuringiensis*: a story of a successful bioinsecticide. *Insect Biochem. Mol. Biol.* 41 423–431. 10.1016/j.ibmb.2011.02.006 21376122 PMC3689885

[B8] Cagirici H. B. Sen T. Z. Budak H. (2021). mirMachine: a one-stop shop for plant miRNA annotation. *J. Vis. Exp.* 171:e62430. 10.3791/62430 33999024

[B9] Calles-Torrez V. Knodel J. J. Boetel M. A. French B. W. Fuller B. W. Ransom J. K. (2019). Field-evolved resistance of northern and western corn rootworm (Coleoptera: Chrysomelidae) populations to corn hybrids expressing single and pyramided Cry3Bb1 and Cry34/35Ab1 Bt proteins in North Dakota. *J. Econ. Entomol.* 112 1875–1886. 10.1093/jee/toz111 31114868

[B10] Chen E. H. Duan J. Y. Song W. Wang D. X. Tang P. A. (2021). RNA-seq analysis reveals mitochondrial and cuticular protein genes are associated with phosphine resistance in the Rusty Grain Beetle (Coleoptera:Laemophloeidae). *J. Econ. Entomol.* 114 440–453. 10.1093/jee/toaa273 33346362

[B11] Chen X. Rosbash M. (2016). Mir-276a strengthens *Drosophila* circadian rhythms by regulating *timeless* expression. *Proc. Natl. Acad. Sci. U.S.A.* 113 E2965–E2972. 10.1073/pnas.1605837113 27162360 PMC4889414

[B12] Cristino A. S. Barchuk A. R. Freitas F. C. Narayanan R. K. Biergans S. D. Zhao Z. (2014). Neuroligin-associated microRNA-932 targets actin and regulates memory in the honeybee. *Nat. Commun.* 5:5529. 10.1038/ncomms6529 25409902

[B13] Cullen B. R. (2004). Transcription and processing of human microRNA precursors. *Mol. Cell* 16 861–865. 15610730 10.1016/j.molcel.2004.12.002

[B14] Dubey S. K. Shrinet J. Sunil S. (2019). *Aedes aegypti* microRNA, miR-2944b-5p interacts with 3’UTR of chikungunya virus and cellular target vps-13 to regulate viral replication. *PLoS Negl. Trop. Dis.* 13:e0007429. 10.1371/journal.pntd.0007429 31166953 PMC6576790

[B15] Etebari K. Afrad M. H. Tang B. Silva R. Furlong M. J. Asgari S. (2018). Involvement of microRNA miR-2b-3p in regulation of metabolic resistance to insecticides in *Plutella xylostella*. *Insect Mol. Biol.* 27 478–491. 10.1111/imb.12387 29573306

[B16] Finn R. D. Bateman A. Clements J. Coggill P. Eberhardt R. Y. Eddy S. R. (2014). Pfam: the protein families database. *Nucleic Acids Res.* 42 D222–D230. 10.1093/nar/gkt1223 24288371 PMC3965110

[B17] Fricke C. Green D. Smith D. Dalmay T. Chapman T. (2014). MicroRNAs influence reproductive responses by females to male sex peptide in *Drosophila melanogaster*. *Genetics* 198 1603–1619. 10.1534/genetics.114.167320 25245794 PMC4256774

[B18] Friedlander M. R. Mackowiak S. D. Li N. Chen W. Rajewsky N. (2012). miRDeep2 accurately identifies known and hundreds of novel microRNA genes in seven animal clades. *Nucleic Acids Res.* 40 37–52. 10.1093/nar/gkr688 21911355 PMC3245920

[B19] Furlong M. J. Wright D. J. Dosdall L. M. (2013). Diamondback moth ecology and management: problems, progress, and prospects. *Annu. Rev. Entomol.* 58 517–541. 10.1146/annurev-ento-120811-153605 23020617

[B20] Goodwin P. R. Meng A. Moore J. Hobin M. Fulga T. A. Van Vactor D. (2018). MicroRNAs regulate sleep and sleep homeostasis in *Drosophila*. *Cell Rep.* 23 3776–3786. 10.1016/j.celrep.2018.05.078 29949763 PMC6091868

[B21] Guo Q. Huang Y. Zou F. Liu B. Tian M. Ye W. (2017). The role of miR-2∼13∼71 cluster in resistance to deltamethrin in *Culex pipiens pallens*. *Insect Biochem. Mol. Biol.* 84 15–22. 10.1016/j.ibmb.2017.03.006 28342977

[B22] Guo Z. Kang S. Sun D. Gong L. Zhou J. Qin J. (2020). MAPK-dependent hormonal signaling plasticity contributes to overcoming *Bacillus thuringiensis* toxin action in an insect host. *Nat. Commun.* 11:3003. 10.1038/s41467-020-16608-8 32532972 PMC7293236

[B23] He J. Chen Q. Wei Y. Jiang F. Yang M. Hao S. (2016). MicroRNA-276 promotes egg-hatching synchrony by up-regulating *brm* in locusts. *Proc. Natl. Acad. Sci. U.S.A* 113 584–589. 10.1073/pnas.1521098113 26729868 PMC4725505

[B24] Janmaat A. F. Myers J. (2003). Rapid evolution and the cost of resistance to *Bacillus thuringiensis* in greenhouse populations of cabbage loopers, *Trichoplusia ni*. *Proc. Biol. Sci.* 270 2263–2270. 10.1098/rspb.2003.2497 14613613 PMC1691497

[B25] Kanehisa M. Goto S. Kawashima S. Okuno Y. Hattori M. (2004). The KEGG resource for deciphering the genome. *Nucleic Acids Res.* 32 D277–D280. 10.1093/nar/gkh063 14681412 PMC308797

[B26] Kitatani Y. Tezuka A. Hasegawa E. Yanagi S. Togashi K. Tsuji M. (2020). *Drosophila* miR-87 promotes dendrite regeneration by targeting the transcriptional repressor Tramtrack69. *PLoS Genet.* 16:e1008942. 10.1371/journal.pgen.1008942 32764744 PMC7439810

[B27] Koonin E. V. Fedorova N. D. Jackson J. D. Jacobs A. R. Krylov D. M. Makarova K. S. (2004). A comprehensive evolutionary classification of proteins encoded in complete eukaryotic genomes. *Genome Biol.* 5:R7. 10.1186/gb-2004-5-2-r7 14759257 PMC395751

[B28] Lee Y. Kim M. Han J. Yeom K. H. Lee S. Baek S. H. (2004). MicroRNA genes are transcribed by RNA polymerase II. *EMBO J.* 23 4051–4060. 10.1038/sj.emboj.7600385 15372072 PMC524334

[B29] Lei Z. Lv Y. Wang W. Guo Q. Zou F. Hu S. (2014). MiR-278-3p regulates pyrethroid resistance in *Culex pipiens pallens*. *Parasitol. Res.* 114 699–706. 10.1007/s00436-014-4236-7 25420996 PMC4304972

[B30] Lewis B. P. Burge C. B. Bartel D. P. (2005). Conserved seed pairing, often flanked by adenosines, indicates that thousands of human genes are microRNA targets. *Cell* 120 15–20. 10.1016/j.cell.2004.12.035 15652477

[B31] Li H. Wei X. Ding T. Chu D. (2018). Genome-wide profiling of *Cardinium*-responsive microRNAs in the exotic whitefly, *Bemisia tabaci* (Gennadius) biotype Q. *Front. Physiol.* 9:1580. 10.3389/fphys.2018.01580 30483149 PMC6241202

[B32] Li R. Huang Y. Zhang Q. Zhou H. Jin P. Ma F. (2019). The miR-317 functions as a negative regulator of Toll immune response and influences *Drosophila* survival. *Dev. Comp. Immunol.* 95 19–27. 10.1016/j.dci.2019.01.012 30708026

[B33] Li R. Zhou H. Jia C. Jin P. Ma F. (2020). *Drosophila* Myc restores immune homeostasis of Imd pathway via activating miR-277 to inhibit imd/Tab2. *PLoS Genet.* 16:e1008989. 10.1371/journal.pgen.1008989 32810129 PMC7455005

[B34] Li S. Xu X. Zheng Z. Zheng J. Shakeel M. Jin F. (2019). MicroRNA expression profiling of *Plutella xylostella* after challenge with *B. thuringiensis*. *Dev. Comp. Immunol.* 93 115–124. 10.1016/j.dci.2018.12.008 30582949

[B35] Li X. Guo L. Zhou X. Gao X. Liang P. (2015). MiRNAs regulated overexpression of ryanodine receptor is involved in chlorantraniliprole resistance in *Plutella xylostella* (L.). *Sci. Rep.* 5:14095. 10.1038/srep14095 26370154 PMC4572936

[B36] Li X. Ren X. Liu Y. Smagghe G. Liang P. Gao X. (2020). MiR-189942 regulates fufenozide susceptibility by modulating ecdysone receptor isoform B in *Plutella xylostella* (L.). *Pestic. Biochem. Physiol.* 163 235–240. 10.1016/j.pestbp.2019.11.021 31973863

[B37] Li Z. Feng X. Liu S. S. You M. Furlong M. J. (2016). Biology, ecology, and management of the diamondback moth in China. *Annu. Rev. Entomol.* 61 277–296. 10.1146/annurev-ento-010715-023622 26667272

[B38] Lim D. H. Lee S. Choi M. S. Han J. Y. Seong Y. Na D. (2020). The conserved microRNA miR-8-3p coordinates the expression of V-ATPase subunits to regulate ecdysone biosynthesis for *Drosophila* metamorphosis. *FASEB J.* 34 6449–6465. 10.1096/fj.201901516R 32196731

[B39] Lim D. H. Lee S. Han J. Y. Choi M. S. Hong J. S. Lee Y. S. (2019). MicroRNA miR-252 targets *mbt* to control the developmental growth of *Drosophila*. *Insect Mol. Biol.* 28 444–454. 10.1111/imb.12562 30582233

[B40] Ling L. Kokoza V. A. Zhang C. Aksoy E. Raikhel A. S. (2017). MicroRNA-277 targets *insulin-like peptides 7* and *8* to control lipid metabolism and reproduction in *Aedes aegypti* mosquitoes. *Proc. Natl. Acad. Sci. U.S.A.* 114 E8017–E8024. 10.1073/pnas.1710970114 28874536 PMC5617303

[B41] Liu B. Tian M. Guo Q. Ma L. Zhou D. Shen B. (2016). MiR-932 regulates pyrethroid resistance in *Culex pipiens pallens* (Diptera: Culicidae). *J. Med. Entomol.* 53 1205–1210. 10.1093/jme/tjw083 27313166 PMC5013817

[B42] Liu J. Ling Z. Wang J. Xiang T. Xu L. Gu C. (2021). In vitro transcriptomes analysis identifies some special genes involved in pathogenicity difference of the *Beauveria bassiana* against different insect hosts. *Microb. Pathog.* 154:104824. 10.1016/j.micpath.2021.104824 33691180

[B43] Liu N. (2015). Insecticide resistance in mosquitoes: impact, mechanisms, and research directions. *Annu. Rev. Entomol.* 60 537–559. 10.1146/annurev-ento-010814-020828 25564745

[B44] Liu S. Xia Q. Zhao P. Cheng T. Hong K. Xiang Z. (2007). Characterization and expression patterns of let-7 microRNA in the silkworm (*Bombyx mori*). *BMC Dev. Biol.* 7:88. 10.1186/1471-213X-7-88 17651473 PMC1976426

[B45] Liu Z. Fu S. Ma X. Baxter S. W. Vasseur L. Xiong L. (2020a). Resistance to *Bacillus thuringiensis* Cry1Ac toxin requires mutations in two *Plutella xylostella* ATP-binding cassette transporter paralogs. *PLoS Pathog.* 16:e1008697. 10.1371/journal.ppat.1008697 32776976 PMC7446926

[B46] Liu Z. Ling L. Xu J. Zeng B. Huang Y. Shang P. (2018). MicroRNA-14 regulates larval development time in *Bombyx mori*. *Insect Biochem. Mol. Biol.* 93 57–65. 10.1016/j.ibmb.2017.12.009 29288754

[B47] Liu Z. Xu J. Ling L. Luo X. Yang D. Yang X. (2020b). miR-34 regulates larval growth and wing morphogenesis by directly modulating ecdysone signaling and cuticle protein in *Bombyx mori*. *RNA Biol.* 17 1342–1351. 10.1080/15476286.2020.1767953 32401141 PMC7549633

[B48] Love M. I. Huber W. Anders S. (2014). Moderated estimation of fold change and dispersion for RNA-seq data with DESeq2. *Genome Biol.* 15:550. 10.1186/s13059-014-0550-8 25516281 PMC4302049

[B49] Lozano J. Montañez R. Belles X. (2015). MiR-2 family regulates insect metamorphosis by controlling the juvenile hormone signaling pathway. *Proc. Natl. Acad. Sci. U.S.A.* 112 3740–3745. 10.1073/pnas.1418522112 25775510 PMC4378413

[B50] Lucas K. J. Roy S. Ha J. Gervaise A. L. Kokoza V. A. Raikhel A. S. (2015). MicroRNA-8 targets the Wingless signaling pathway in the female mosquito fat body to regulate reproductive processes. *Proc. Natl. Acad. Sci. U.S.A.* 112 1440–1445. 10.1073/pnas.1424408112 25605933 PMC4321257

[B51] Luo W. Huang L. X. Qin S. K. Zhang X. Feng Q. L. Gu J. (2020). Multiple microRNAs control ecdysone signaling in the midgut of *Spodoptera litura*. *Insect Sci.* 27 1208–1223. 10.1111/1744-7917.12745 31840397

[B52] Ma K. Li X. Hu H. Zhou D. Sun Y. Ma L. (2017). Pyrethroid-resistance is modulated by miR-92a by targeting *CpCPR4* in *Culex pipiens pallens*. *Comp. Biochem. Phys. B.* 203 20–24. 10.1016/j.cbpb.2016.09.002 27627779 PMC5143170

[B53] Marco A. Hooks K. Griffiths-Jones S. (2012). Evolution and function of the extended miR-2 microRNA family. *RNA Biol.* 9 242–248. 10.4161/rna.19160 22336713 PMC3384581

[B54] Meuti M. E. Bautista-Jimenez R. Reynolds J. A. (2018). Evidence that microRNAs are part of the molecular toolkit regulating adult reproductive diapause in the mosquito, *Culex pipiens*. *PLoS One* 13:e0203015. 10.1371/journal.pone.0203015 30496183 PMC6264513

[B55] Meyers B. C. Axtell M. J. Bartel B. Bartel D. P. Baulcombe D. Bowman J. L. (2008). Criteria for annotation of plant MicroRNAs. *Plant Cell* 20 3186–3190. 10.1105/tpc.108.064311 19074682 PMC2630443

[B56] Mohebbi M. Ding L. Malmberg R. L. Cai L. (2021). Human MicroRNA target prediction via multi-hypotheses learning. *J. Comput. Biol.* 28 117–132. 10.1089/cmb.2020.0227 33232617 PMC7910415

[B57] Niu Y. Liu Z. Nian X. Xu X. Zhang Y. (2019). MiR-210 controls the evening phase of circadian locomotor rhythms through repression of *Fasciclin 2*. *PLoS Genet.* 15:e1007655. 10.1371/journal.pgen.1007655 31356596 PMC6687186

[B58] Palma L. Munoz D. Berry C. Murillo J. Caballero P. (2014). *Bacillus thuringiensis* toxins: an overview of their biocidal activity. *Toxins (Basel)* 6 3296–3325. 10.3390/toxins6123296 25514092 PMC4280536

[B59] Pardo-Lopez L. Soberon M. Bravo A. (2013). *Bacillus thuringiensis* insecticidal three-domain Cry toxins: mode of action, insect resistance and consequences for crop protection. *FEMS Microbiol. Rev.* 37 3–22. 10.1111/j.1574-6976.2012.00341.x 22540421

[B60] Peng T. Pan Y. Gao X. Xi J. Zhang L. Ma K. (2016). Reduced abundance of the *CYP6CY3*-targeting let-7 and miR-100 miRNAs accounts for host adaptation of *Myzus persicae* nicotianae. *Insect Biochem. Mol. Biol.* 75 89–97. 10.1016/j.ibmb.2016.06.002 27318250

[B61] Puoliväli T. Palva S. Palva J. M. (2020). Influence of multiple hypothesis testing on reproducibility in neuroimaging research: a simulation study and Python-based software. *J. Neurosci. Methods* 337:108654. 10.1016/j.jneumeth.2020.108654 32114144

[B62] Qin S. Zhang S. Sun X. Kong Y. Hou C. Li M. (2021). Transcriptome reveal the response to Cry1Ac toxin in susceptible *Bombyx mori*. *Arch Insect Biochem. Physiol.* 107:e21794. 10.1002/arch.21794 33948968

[B63] Raymond B. Johnston P. R. Nielsen-LeRoux C. Lereclus D. Crickmore N. (2010). *Bacillus thuringiensis*: an impotent pathogen? *Trends Microbiol.* 18 189–194. 10.1016/j.tim.2010.02.006 20338765

[B64] Rehmsmeier M. Steffen P. Hochsmann M. Giegerich R. (2004). Fast and effective prediction of microRNA/target duplexes. *Bioinformatics* 10 1507–1517. 10.1261/rna.5248604 15383676 PMC1370637

[B65] Reynolds J. A. Nachman R. J. Denlinger D. L. (2019). Distinct microRNA and mRNA responses elicited by ecdysone, diapause hormone and a diapause hormone analog at diapause termination in pupae of the corn earworm, *Helicoverpa zea*. *Gen. Comp. Endocrinol.* 278 68–78. 10.1016/j.ygcen.2018.09.013 30243885

[B66] Robertson H. M. Waterhouse R. M. Walden K. K. O. Ruzzante L. Reijnders M. Coates B. S. (2018). Genome sequence of the wheat stem sawfly, *Cephus cinctus*, representing an early-branching lineage of the hymenoptera, illuminates evolution of hymenopteran chemoreceptors. *Genome Biol. Evol.* 10 2997–3011. 10.1093/gbe/evy232 30335145 PMC6250288

[B67] Shakeel M. Xu X. Xu J. Li S. Yu J. Zhou X. (2018). Genome-wide identification of destruxin a-responsive immunity-related microRNAs in diamondback moth, *Plutella xylostella*. *Front. Immunol.* 9:185. 10.3389/fimmu.2018.00185 29472927 PMC5809476

[B68] Shan S. Wang S. N. Song X. Khashaveh A. Lu Z. Y. Dhiloo K. H. (2020). Characterization and target gene analysis of microRNAs in the antennae of the parasitoid wasp *Microplitis mediator*. *Insect Sci.* 28 1033–1048. 10.1111/1744-7917.12832 32496619

[B69] Shen Z. J. Liu Y. J. Zhu F. Cai L. M. Liu X. M. Tian Z. Q. (2020). MicroRNA-277 regulates dopa decarboxylase to control larval-pupal and pupal-adult metamorphosis of *Helicoverpa armigera*. *Insect Biochem. Mol. Biol.* 122:103391. 10.1016/j.ibmb.2020.103391 32360955

[B70] Singh C. P. (2020). Role of microRNAs in insect-baculovirus interactions. *Insect Biochem. Mol. Biol* 127:103459. 10.1016/j.ibmb.2020.103459 32961323

[B71] Singh C. P. Singh J. Nagaraju J. (2014). Bmnpv-miR-3 facilitates BmNPV infection by modulating the expression of viral P6.9 and other late genes in *Bombyx mori*. *Insect Biochem. Mol. Biol.* 49 59–69. 10.1016/j.ibmb.2014.03.008 24698834

[B72] Song J. Li W. Zhao H. Zhou S. (2019). Clustered miR-2, miR-13a, miR-13b and miR-71 coordinately target *Notch* gene to regulate oogenesis of the migratory locust *Locusta migratoria*. *Insect Biochem. Mol. Biol.* 106 39–46. 10.1016/j.ibmb.2018.11.004 30453026

[B73] Su J. Wang G. Li C. Xing D. Yan T. Zhu X. (2019). Screening for differentially expressed miRNAs in *Aedes albopictus* (Diptera: Culicidae) exposed to DENV-2 and their effect on replication of DENV-2 in C6/36 cells. *Parasit Vectors* 12:44. 10.1186/s13071-018-3261-2 30658692 PMC6339288

[B74] Sun K. Jee D. de Navas L. F. Duan H. Lai E. C. (2015). Multiple in vivo biological processes are mediated by functionally redundant activities of *Drosophila* mir-279 and mir-996. *PLoS Genet.* 11:e1005245. 10.1371/journal.pgen.1005245 26042831 PMC4456407

[B75] Sun Q. Guo H. Xia Q. Jiang L. Zhao P. (2020). Transcriptome analysis of the immune response of silkworm at the early stage of *Bombyx mori* bidensovirus infection. *Dev. Comp. Immunol.* 106:103601. 10.1016/j.dci.2019.103601 31899306

[B76] Sun X. H. Xu N. Xu Y. Zhou D. Sun Y. Wang W. J. (2019). A novel miRNA, miR-13664, targets *CpCYP314A1* to regulate deltamethrin resistance in *Culex pipiens pallens*. *Parasitology* 146 197–205. 10.1017/S0031182018001002 29966536 PMC6318076

[B77] Tabashnik B. E. Carrière Y. (2017). Surge in insect resistance to transgenic crops and prospects for sustainability. *Nat. Biotechnol.* 35 926–935. 10.1038/nbt.3974 29020006

[B78] Tabashnik B. E. Carrière Y. (2019). Global patterns of resistance to Bt crops highlighting Pink Bollworm in the United States, China, and India. *J. Econ. Entomol.* 112 2513–2523. 10.1093/jee/toz173 31254345

[B79] Tatusov R. L. Galperin M. Y. Natale D. A. Koonin E. V. (2000). The COG database: a tool for genome-scale analysis of protein functions and evolution. *Nucleic Acids Res.* 28 33–36. 10.1093/nar/28.1.33 10592175 PMC102395

[B80] Tian M. Liu B. Hu H. Li X. Guo Q. Zou F. (2016). MiR-285 targets P450 (*CYP6N23*) to regulate pyrethroid resistance in *Culex pipiens pallens*. *Parasitol. Res.* 115 4511–4517. 10.1007/s00436-016-5238-4 27651043

[B81] Vaschetto L. M. Beccacece H. M. (2019). The emerging importance of noncoding RNAs in the insecticide tolerance, with special emphasis on *Plutella xylostella* (Lepidoptera: Plutellidae). *WIREs RNA* 10:e1539. 10.1002/wrna.1539 31045325

[B82] Wei X. Zheng C. Peng T. Pan Y. Xi J. Chen X. (2016). miR-276 and miR-3016-modulated expression of acetyl-CoA carboxylase accounts for spirotetramat resistance in *Aphis gossypii* Glover. *Insect Biochem. Mol. Biol.* S0965-1748 30158–30158. 10.1016/j.ibmb.2016.10.011 27989834

[B83] Wen M. Shen Y. Shi S. Tang T. (2012). miREvo: an integrative microRNA evolutionary analysis platform for next-generation sequencing experiments. *BMC Bioinformatics* 13:140. 10.1186/1471-2105-13-140 22720726 PMC3410788

[B84] Wu K. M. Lu Y. H. Feng H. Q. Jiang Y. Y. Zhao J. Z. (2008). Suppression of cotton bollworm in multiple crops in China in areas with Bt toxin-containing cotton. *Science* 321 1676–1678. 10.1126/science.1160550 18801998

[B85] Wu P. Jiang X. Sang Q. Annan E. Cheng T. Guo X. (2017). Inhibition of miR-274-3p increases BmCPV replication by regulating the expression of BmCPV NS5 gene in *Bombyx mori*. *Virus Genes* 53 643–649. 10.1007/s11262-017-1466-7 28493152

[B86] Wu P. Shang Q. Dweteh O. A. Huang H. Zhang S. Zhong J. (2019). Over expression of bmo-miR-2819 suppresses BmNPV replication by regulating the BmNPV ie-1 gene in *Bombyx mori*. *Mol. Immunol.* 109 134–139. 10.1016/j.molimm.2019.03.013 30947109

[B87] Xu L. N. Ling Y. H. Wang Y. Q. Wang Z. Y. Hu B. J. Zhou Z. Y. (2015). Identification of differentially expressed microRNAs between *Bacillus thuringiensis* Cry1Ab-resistant and -susceptible strains of *Ostrinia furnacalis*. *Sci. Rep.* 5:15461. 10.1038/srep15461 26486179 PMC4614346

[B88] Xu X. Yang J. Harvey-Samuel T. Huang Y. Asad M. Chen W. (2020). Identification and characterization of the *vasa* gene in the diamondback moth, *Plutella xylostella*. *Insect Biochem. Mol. Biol.* 122:103371. 10.1016/j.ibmb.2020.103371 32283279

[B89] Xu X. Zhu H. Yang F. Wu C. Jiang C. Yu W. (2019). Bmo-miR-79 downregulates the expression of *BmEm4* in the silkworm, *Bombyx mori*. *Gene* 690 113–119. 10.1016/j.gene.2018.12.034 30593917

[B90] Yan H. Zhou Y. Liu Y. Deng Y. Chen X. (2014). miR-252 of the Asian tiger mosquito *Aedes albopictus* regulates dengue virus replication by suppressing the expression of the dengue virus envelope protein. *J. Med. Virol.* 86 1428–1436. 10.1002/jmv.23815 25025105

[B91] Zhang Q. Dou W. Song Z.-H. Jin T.-J. Yuan G.-R. De Schutter K. (2020). Identification and profiling of *Bactrocera dorsalis* microRNAs and their potential roles in regulating the developmental transitions of egg hatching, molting, pupation and adult eclosion. *Insect Biochem. Mol. Biol.* 127:103475. 10.1016/j.ibmb.2020.103475 33059019

[B92] Zhao J. Z. Li Y. X. Collins H. L. Shelton A. M. (2002). Examination of the F2 screen for rare resistance alleles to *Bacillus thuringiensis* toxins in the diamondback moth (Lepidoptera: Plutellidae). *J. Econ. Entomol.* 95 14–21. 10.1603/0022-0493-95.1.14 11942749

[B93] Zhu B. Li X. Liu Y. Gao X. Liang P. (2017). Global identification of microRNAs associated with chlorantraniliprole resistance in diamondback moth *Plutella xylostella* (L.). *Sci. Rep.* 7:40713. 10.1038/srep40713 28098189 PMC5241650

[B94] Zhu B. Sun X. Nie X. Liang P. Gao X. (2019). MicroRNA-998-3p contributes to Cry1Ac-resistance by targeting *ABCC2* in lepidopteran insects. *Insect Biochem. Mol. Biol.* 117:103283. 10.1016/j.ibmb.2019.103283 31759051

