# Supplementary material for: Profiling of MicroRNAs in Midguts of Plutella xylostella Provides Novel Insights Into the Bacillus thuringiensis Resistance
Source: Front Genet. 2021 Sep 8;12:739849. doi: 10.3389/fgene.2021.739849 (PMC8455949; doi:10.3389/fgene.2021.739849)
Supplement: Supplementary file 1 [file Table_1.DOCX]

**Table S1 Sequences of primers used for RT-qPCR of differentially expressed miRNAs**

| miRNA | Sequence (5’-3’) |
| --- | --- |
| Novel_miR_210 | CG UUGUGACGUAGGAUUGUCAAUA |
| Novel-miR-274 | UCAGUCUCUGUAUUCUCCCUUCA |
| Novel-miR-288 | CG CUAAUCCUCCAUAGACUCUAGAUU |
| Novel-miR-48 | UAGCACCGUAGCAUUGAAGU |
| Novel-miR-240 | CG UCCUCAAUAUCAUAUUCCUCGC |
| Novel-miR-97 | GG CCUAGCUAGUAAUUAAUCAUCGA |
| Novel-miR-25 | CAGAGAAACCUGACCCCCCUCC |
| Pxy-miR-8522 | AUUUGCCGAAGGUUCUGAUACC |
| Novel-miR-237 | UUUCCACCAGAGAUGUGCUAUG |
| Novel-miR-225 | CGC UUAAAUCGAGAUUUGACACU |
| Novel-miR-270 | CG AAACAUCCAUGUAAACAUCCUGA |
| Novel-miR-116 | CC UACCUAAGCAGGAACUGUUC |

The underlines under the sequences represent bases added to adjust the Tm value of primers.
