# Supplementary material for: Profiling of MicroRNAs in Midguts of Plutella xylostella Provides Novel Insights Into the Bacillus thuringiensis Resistance
Source: Front Genet. 2021 Sep 8;12:739849. doi: 10.3389/fgene.2021.739849 (PMC8455949; doi:10.3389/fgene.2021.739849)
Supplement: Supplementary file 2 [file Table_2.DOCX]

**Table S2 Families of identified miRNAs**

| Family | Identified miRNA | Family | Identified miRNA |
| --- | --- | --- | --- |
| let-7 | novel-miR-133  novel-miR-250 | miR-3689 | novel-miR-263 |
| lin-4 | novel-miR-356 | miR-375 | novel-miR-121 |
| miR-1 | novel-miR-176  mse-miR-1b | miR-430 | novel-miR-355  novel-miR-41 |
| miR-10 | novel-miR-160  novel-miR-206 | miR-4529 | novel-miR-324  novel-miR-351 |
| miR-11 | novel-miR-29 | miR-46 | novel-miR-360  novel-miR-89 |
| miR-1175 | novel-miR-235  novel-miR-34 | miR-4680 | novel-miR-215  novel-miR-243  novel-miR-153  novel-miR-342 |
| miR-130 | novel-miR-336 | miR-4864 | novel-miR-138  novel-miR-25  novel-miR-128  novel-miR-272  novel-miR-151  novel-miR-117  novel-miR-313  novel-miR-208  novel-miR-102  novel-miR-83  novel-miR-62  novel-miR-92 |
| miR-137 | novel-miR-327 | miR-4891 | novel-miR-183 |
| miR-14 | novel-miR-93  novel-miR-125 | miR-541 | novel-miR-277  novel-miR-123  novel-miR-14 |
| miR-154 | novel-miR-169  novel-miR-289 | miR-587 | novel-miR-237 |
| miR-184 | novel-miR-28  pxy-miR-184 | miR-5904 | novel-miR-196 |
| miR-185 | novel-miR-98  novel-miR-141  novel-miR-166  novel-miR-170  novel-miR-3 | miR-6 | novel-miR-2 |
| miR-190 | novel-miR-186  novel-miR-232 | miR-628 | pxy-miR-8494-3p |
| miR-192 | novel-miR-213 | miR-67 | novel-miR-344 |
| miR-2 | novel-miR-222  novel-miR-219  novel-miR-214  novel-miR-142  novel-miR-253 | miR-7 | novel-miR-252  pxy-miR-7b |
| miR-2024 | novel-miR-69  novel-miR-248  novel-miR-30 | miR-71 | novel-miR-90 |
| miR-203 | novel-miR-11  novel-miR-16 | miR-7371 | pxy-miR-8532-3p |
| miR-216 | pxy-miR-283 | miR-750 | pxy-miR-750 |
| miR-219 | novel-miR-59  novel-miR-159 | miR-76 | novel-miR-364  novel-miR-54 |
| miR-22 | pxy-miR-8493 | miR-7880 | novel-miR-216 |
| miR-25 | novel-miR-337 | miR-7948 | novel-miR-195 |
| miR-252 | pxy-miR-252 | miR-7957 | novel-miR-103 |
| miR-2525 | pxy-miR-2525-3p | miR-8489 | pxy-miR-8489a-5p  pxy-miR-8489a-3p |
| miR-253 | novel-miR-119 | miR-8499 | pxy-miR-8499b |
| miR-263 | novel-miR-10  novel-miR-1 | miR-8510 | pxy-miR-8510a-3p  pxy-miR-8510b-5p  pxy-miR-8510a-8-3p  pxy-miR-8510b-3p  pxy-miR-8510a-5p |
| miR-2733 | novel-miR-39  novel-miR-182  novel-miR-66 | miR-8515 | pxy-miR-8515 |
| miR-274 | pxy-miR-274 | miR-8517 | novel-miR-109  novel-miR-221  novel-miR-189  novel-miR-228  novel-miR-13  novel-miR-32  pxy-miR-8517b  novel-miR-319  novel-miR-302  pxy-miR-8517a |
| miR-2755 | novel-miR-347 | miR-8521 | pxy-miR-8521b  pxy-miR-8521a |
| miR-276 | lmi-miR-276-5p  lmi-miR-276-3p  novel-miR-78 | miR-8524 | pxy-miR-8524-3p  pxy-miR-8524-5p |
| miR-2763 | novel-miR-80 | miR-8535 | pxy-miR-8535-5p  pxy-miR-8535-3p |
| miR-2767 | novel-miR-333  novel-miR-161 | miR-8536 | pxy-miR-8536b-5p  pxy-miR-8536b-3p |
| miR-277 | pxy-miR-277  hme-miR-277  mse-miR-277 | miR-8539 | pxy-miR-8539-3p  pxy-miR-8539-5p |
| miR-278 | novel-miR-217 | miR-8544 | pxy-miR-8544-3p  pxy-miR-8544-5p |
| miR-279 | novel-miR-343  pxy-miR-279a  pxy-miR-279b-5p  pxy-miR-279b-3p  bmo-miR-279c-5p  bmo-miR-279c-3p | miR-87 | novel-miR-199 |
| miR-2796 | novel-miR-285  novel-miR-22 | miR-8908 | novel-miR-255  novel-miR-244  novel-miR-325 |
| miR-282 | novel-miR-43  novel-miR-20 | miR-9 | pxy-miR-9b-5p  novel-miR-290  novel-miR-200  novel-miR-70  pxy-miR-9b-3p  novel-miR-204 |
| miR-2843 | pxy-miR-8513-5p | miR-90 | novel-miR-76 |
| miR-306 | pxy-miR-306 | miR-9128 | novel-miR-124  novel-miR-311 |
| miR-308 | pxy-miR-308 | miR-9191 | novel-miR-110 |
| miR-31 | novel-miR-246 | miR-927 | novel-miR-50 |
| miR-316 | novel-miR-265 | miR-929 | pxy-miR-929 |
| miR-317 | novel-miR-21 | miR-965 | novel-miR-162 |
| miR-33 | novel-miR-55 | miR-970 | novel-miR-104 |
| miR-331 | novel-miR-357 | miR-973 | novel-miR-24  novel-miR-97  novel-miR-118 |
| miR-34 | novel-miR-310  novel-miR-271 | miR-981 | novel-miR-108 |
| miR-iab-4 | bmo-miR-iab-4-5p |  |  |
