# Supplementary material for: Profiling of MicroRNAs in Midguts of Plutella xylostella Provides Novel Insights Into the Bacillus thuringiensis Resistance
Source: Front Genet. 2021 Sep 8;12:739849. doi: 10.3389/fgene.2021.739849 (PMC8455949; doi:10.3389/fgene.2021.739849)
Supplement: Supplementary file 4 [file Table_4.DOCX]

**Table S4 KEGG enrichment of differentially expressed miRNAs target genes**

| ko ID | Pathway | GeneRatio | BgRatio | Enrich factor | *p*-value | *q*-value | Gene number | Gene ID |
| --- | --- | --- | --- | --- | --- | --- | --- | --- |
| ko00970 | Aminoacyl-tRNA biosynthesis | 14.29% | 1.18% | 12.07 | 0.011 | 0.058 | 2 | *Px007475, Px008286* |
| ko04142 | Lysosome | 7.14% | 3.22% | 2.22 | 0.37 | 0.34 | 1 | *Px002146* |
| ko04013 | MAPK signaling pathway - fly | 7.14% | 2.7% | 2.65 | 0.32 | 0.34 | 1 | *Px006958* |
| ko00910 | Nitrogen metabolism | 7.14% | 0.39% | 18.11 | 0.054 | 0.19 | 1 | *Px002493* |
| ko04145 | Phagosome | 7.14% | 2.17% | 3.29 | 0.27 | 0.34 | 1 | *Px003379* |
| ko01212 | Fatty acid metabolism | 7.14% | 1.84% | 3.88 | 0.23 | 0.34 | 1 | *Px013839* |
| ko04392 | Hippo signaling pathway -multiple species | 14.29% | 0.85% | 16.71 | 0.0058 | 0.058 | 2 | *Px016226, Px008940* |
| ko04120 | Ubiquitin mediated proteolysis | 7.14% | 2.76% | 2.59 | 0.33 | 0.34 | 1 | *Px016576* |
| ko00512 | Mucin type O-Glycan biosynthesis | 7.14% | 0.66% | 10.86 | 0.089 | 0.22 | 1 | *Px002006* |
| ko04141 | Protein processing in endoplasmic reticulum | 7.14% | 3.42% | 2.09 | 0.39 | 0.34 | 1 | *Px014239* |
| ko03008 | Ribosome biogenesis in eukaryotes | 7.14% | 1.71% | 4.18 | 0.22 | 0.34 | 1 | *Px009012* |
| ko00534 | Glycosaminoglycan biosynthesis - heparan sulfate / heparin | 7.14% | 0.79% | 9.05 | 0.11 | 0.22 | 1 | *Px013363* |

ko ID: KEGG pathway ID; GeneRatio: the ratio of the number of DE miRNAs target genes to the number of all target genes enriched in this pathway; BgRatio: the ratio of the number of all DE miRNAs target genes to the number of all target genes enriched in the whole pathways; enrich factor: the ratio of the GeneRatio to the BgRatio; *p*-value: the *p*-value after Fisher test; *q*-value: corrected *p*-value by FDR; Gene number: the number of DE miRNAs target genes enriched in this pathway.
